# Psychological distress among workers at a mine

**DOI:** 10.4102/sajpsychiatry.v31i0.2422

**Published:** 2025-06-30

**Authors:** Yolanda Havenga, Michelle Bester

**Affiliations:** 1 Adelaide Tambo School of Nursing Science, Faculty of Science, Tshwane University of Technology, Pretoria, South Africa

**Keywords:** alcohol use disorder inventory, Kessler’s Psychological Distress Scale (K10), mental health, mine workers, psychological distress

## Abstract

**Background:**

Mining industries are high-risk workplaces for psychological distress. Unaddressed psychological distress can lead to accidents, absenteeism and decreased productivity.

**Aim:**

This study sought to determine levels of psychological distress and associated sociodemographic factors among employees working at a mining company in South Africa.

**Setting:**

The study was conducted at four sites, including three underground mines and a head office, across a number of provinces in South Africa.

**Methods:**

A quantitative correlation design was used with 927 respondents recruited through convenience sampling. Data were collected using a questionnaire containing sociodemographic items, the Kessler Psychological Distress Scale (K10) and the Alcohol Use Disorder Identification Test. The response rate was 84%.

**Results:**

Two-thirds (69%) of participants reported no or mild psychological distress, while a third (31%) experienced moderate to severe distress. Women, younger employees, employees at site 1 and those with increased likelihood of alcohol consumption at higher risk levels, were more likely to experience higher levels of psychological distress.

**Conclusion:**

Psychological distress potentially impacts daily functioning for a third of employees, indicating a need for prevention and management interventions addressing personal, workplace and environmental factors.

**Contribution:**

This study identifies critical sociodemographic factors associated with psychological distress among South African mine employees. These factors can inform targeted mental health interventions to improve employees’ mental health, safety and productivity. The findings highlight the need to focus on targeted mental health interventions for women and younger employees and to design interventions that address alcohol use and mental health in an integrated manner.

## Introduction

Mental health conditions have a high global prevalence, with approximately one in eight people in the world living with a mental disorder and one in three people in South Africa who will experience a mental illness during their lifetime.^1,2^ Predominantly attributable to preventable comorbid physical illnesses, people with mental illness have a 12–16 year shorter life expectancy than the general population.^3^ Poor mental health affects a person’s thinking, behaviour, emotions, social and relational well-being, physical health and identity, affecting their capacity to participate in work.^4^ Lost time at work and work losses due to mental illness lead to reduced household earning abilities. The wider societal costs related to unemployment, loss of productivity, loss of skilled workers and reduced tax revenue significantly outweigh the healthcare costs. The estimated cumulative global cost of mental conditions due to lost productivity is 1 trillion U.S. Dollars, with 12 billion working days lost every year due to depression and anxiety.^4^

Physical and psychosocial hazards can affect workers’ mental health adversely and increase the likelihood of the occurrence of a mental illness.^4,5^ Psychosocial hazards affecting mental health include those related to work content, workload, work schedule, control, environment and equipment, organisational culture and function, interpersonal relationships at work, role in the organisation, career development and home–work interface.^4^

Mining industries, predominantly male-dominated workplaces, are high-risk environments for psychological distress attributable to long shift patterns, high job demands, physical risks and working in isolated environments.^5,6,7,8^ Mining environments differ, as open pits, shafts and drift mines exist. The types of jobs at a mine are affected by the type of mine, with shaft mines having underground and surface operations such as processing, maintenance, administration and health and safety roles. Each of these has its unique job demands.

These working conditions have the potential to increase psychological distress, a state of emotional suffering associated with stressors and demands that are difficult to cope with daily life and typically characterised by symptoms of anxiety and depression.^9,10^ Creating safe and healthy workplaces can promote mental health among employees, support recovery and mitigate the risk of mental ill health by minimising workplace hazards.^7^

Therefore, the purpose of the study is to determine the levels of psychological distress and associated sociodemographic variables among employees at a mining company in South Africa.

## Research methods and design

### Study design

A quantitative correlational design using a cross-sectional survey was implemented.

### Setting

The study was conducted at four sites of a mining company: three mines with underground operations and the head office, situated across a number of provinces in South Africa.

### Study population and sampling strategy

**FIGURE 1:**
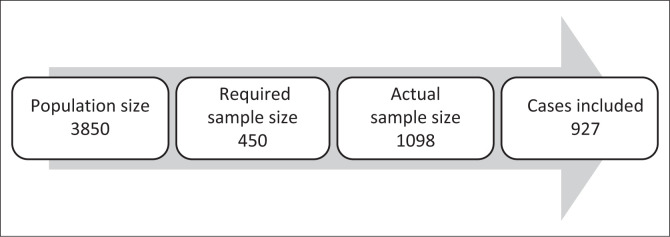
Flow diagram of population and sample sizes.

The population consisted of underground mine workers, plant workers (those processing and refining extracted minerals and maintaining equipment) and workers in office-bound positions, including managers and administrators. Nonprobability convenience sampling was used to sample 1098 participants from a total population of 3850 employees. At a 5% margin of error, a 95% confidence level and a 50% response distribution, the minimum recommended sample size for a population of 3850 employees was 350 participants.^11^ Because seven predictors were included, the minimum sample size was set as 450. Based on the questionnaires completed (*n* = 1098) and cases included (*n* = 927), the response rate is 84%, higher than the *American Journal of Pharmaceutical Education*’s 60% goal for survey responses.^12^ Figure 1 provides a flow diagram of the population size, the required sample size, the actual sample size and the complete cases included in the final analysis.

After managers’ briefing, employees were approached when reporting for work or during lunch times; the details of the study were explained to them, and informed consent was obtained. This recruitment was done in person, followed by online recruitment of those who could not be reached conveniently. Participants working at the surface and underground at various work stations and for all four sites were sampled.

### Data collection

Data collection took place from March to September 2023, initially by four fieldworkers at each research site for a total of 9 days, followed by the distribution of the online link to the questionnaire for another month. Of all the questionnaires completed, 26 were completed online, and they were all from site 4, and readily had access to emails. The nature of employees’ work at sites 1, 2 and 3 made using paper-based questionnaires more accessible. Paper-based questionnaires were self-administered, with fieldworkers providing clarity when required. The questionnaire administered consisted of three sections.

#### Section A: Sociodemographic characteristics

Personal, work-related and alcohol-use-related data were collected. The covariables included gender, age, relationship status, work environment, work schedule, site of employment and alcohol use patterns.^5,13^

#### Section B: Psychological distress

The Kessler Psychological Distress Scale (K10) was used to measure general psychological distress as it has been proven effective in measuring peoples’ current level of mental health.^14^ The scale had 10 questions, and a Likert scale with five response levels (‘none’ to ‘all of the time’). Low scores indicate low levels of psychological distress and high scores indicate elevated levels of psychological distress. The K10 scale has ordinal categories for low (10–19), mild (20–24), moderate (25–29) and high (≥ 30) and as binary variables low to mild (0–24) and moderate to high (25 to ≥ 30). The Cronbach Alpha for Section B was 0.924, which shows a high level of reliability.^15^

#### Section C: Alcohol use disorder identification test

The Alcohol use disorder identification test (AUDIT) is a screening tool developed by the World Health Organization (WHO) to assess for unhealthy alcohol use, consisting of 10 items with a 5-item Likert scale. The 10 questions cover three domains: hazardous alcohol use, dependence symptoms and harmful alcohol use. Scoring groups the participants’ alcohol use risk as low, risky or hazardous and elevated risk or harmful level.^16^ The Cronbach Alpha for Section C was 0.855, which shows a high level of reliability.^15^

The surveys took 10–15 min to complete and were available in English, Sesotho and isiZulu. The questionnaire was piloted and required no adjustment; however, the piloted data were not included in the final sample size.

### Data analysis

Data were captured in an Excel spreadsheet, and the accuracy of capturing was rechecked. Personal, workplace and alcohol use variables were analysed using descriptive statistics and described as frequencies and percentages using a Statistical Analysis System (SAS). Chi-squared tests were used to examine the bivariate association of categorical predictors (including participants’ personal, work-related and alcohol-use-related characteristics) with the dichotomous outcome variables for psychological distress (combined low to moderate and high to very high) of the K10 scores.^7^ Multivariate logistical regression analysis of the association between the sociodemographic characteristics and level of psychological distress was done for all complete cases, and the results were presented as coefficients with a 95% confidence interval (CI) and a level of significance of *p* < 0.05. All variables were included to ensure that the model adjusted for potential confounding variables, aligned with theoretical expectations, and accurately represented the complex interplay of factors influencing psychological distress.

### Ethical considerations

Ethical clearance to conduct this study was obtained from the Tshwane University of Technology Research Ethics Committee (REC) (No. REC/2023/01/002), and the mining company’s gatekeeper approved the study. By reading the information leaflet and completing the questionnaire, respondents consented to participate in the study. Information and consent letters were available in three languages. Fieldworkers signed a confidentiality agreement, and all identifying information about respondents was removed to anonymise questionnaires and reports. The most secure version of the online survey, SurveyMonkey Enterprise ® was used. Hard copies of the questionnaires are stored at the university for safekeeping and will be destroyed after 3 years.

## Results

### Sample characteristics

A total of 927 respondents completed all components of the survey’s demographic and psychological distress sections. The sociodemographic characteristics based on personal, workplace and alcohol use variables are indicated in Table 1. The majority of participants were male (*n* = 654, 70.6%), between 25 and 44 years of age (*n* = 607, 65.4%), and were in some form of a relationship (*n* = 765, 82.5%). In 2023, 21% of the workforce were women in the mining company. In terms of the work environment, the majority of respondents worked at the surface (office and plant) (*n* = 568, 61.2%), office hours (*n* = 524, 56.5%) and worked at mine site 2 (*n* = 567, 61.2%). Self-reported alcohol use patterns indicated that 603 (65%) were at low risk, 174 (18.8%) were at medium risk, 45 (4.9%) were at high risk, and 44 (4.7%) indicated addiction to be likely. Sixty-one (6.6%) of the participants did not complete the AUDIT section of the questionnaire.

**TABLE 1:** Bivariate association between psychological distress and characteristics of the sample.

Variable	Psychological distress dichotomised				Total (*N* = 927)		*p*
	Low level of distress *N* = 658 (71%)		High level of distress *N* = 269 (29%)				
	*n*	%	*n*	%	*n*	%	
**Gender**							
Male	481.0	73.5	173.0	26.5	654	70.6	0.008 *
Female	177.0	64.8	96.0	35.2	273	29.4	
**Age (years)**							
24	21.0	67.7	10.0	32.3	31	3.3	0.211
25–34	208.0	70.3	88.0	29.7	296	31.9	
35–44	211.0	67.8	100.0	32.2	311	33.5	
45–54	147.0	72.8	55.0	27.2	202	21.8	
55–65	69.0	82.1	15.0	17.9	84	9.1	
> 65	2.0	66.7	1.0	33.3	3	0.3	
**Relationship status**							
Single	78.0	72.2	30.0	27.8	108	11.7	0.213
In a relationship (Living apart)	133.0	64.9	72.0	35.1	205	22.1	
In a relationship (Living together)	120.0	75.9	38.0	24.1	158	17.0	
Separated	11.0	61.1	7.0	38.9	18	1.9	
Widowed	12.0	75.0	4.0	25.0	16	1.7	
Married	292.0	72.6	110.0	27.4	402	43.4	
Divorced	12.0	60.0	8.0	40.0	20	2.2	
**Work area**							
Office	219.0	71.3	88.0	28.7	307	33.1	0.869
Plant	182.0	69.7	79.0	30.3	261	28.2	
Underground	257.0	71.6	102.0	28.4	359	38.7	
**Work schedule**							
Office hours	372.0	71.0	152.0	29.0	524	56.5	0.993
Shift hours	286.0	71.0	117.0	29.0	403	43.5	
**Mine site**							
Site 1	188.0	63.5	108.0	36.5	296	31.9	0.002*
Site 2	428.0	75.5	139.0	24.5	567	61.2	
Site 3	26.0	68.4	12.0	31.6	38	4.1	
Site 4	16.0	61.5	10.0	38.5	26	2.8	
**Alcohol use**							
Low risk	461.0	76.5	142.0	23.5	603	65.0	< 0.001*
Medium risk	117.0	67.2	57.0	32.8	174	18.8	
High risk	24.0	53.3	21.0	46.7	45	4.9	
Addiction likely	18.0	40.9	26.0	59.1	44	4.7	
Unknown use	38.0	62.3	23.0	37.7	61	6.6	

Note: *p*-value: Probability > chi^2^.

Significant: *p < 0.05. Chi-square analyses revealed several significant associations between psychological distress levels and personal, work and alcohol use variables.

### Psychological distress

Table 1 indicates the levels of psychological distress and the bivariate association between the covariates and the psychological distress.

Among the 927 respondents, over two-thirds (*n* = 658, 71%) had no or mild psychological distress, and 29% (*n* = 269) experienced moderate to severe psychological distress.

A statistically significant association was found between gender and psychological distress (*p* = 0.008). Males were more likely to report low distress levels (73.5%), with a lower proportion (26.5%) reporting high levels of distress. Females were more likely than men, to report high levels of distress (35.2%) and 64.8% reporting low levels of distress.

The distribution of psychological distress across different mine sites showed a statistically significant association (*p* = 0.002). Site 2, which accounted for the largest portion of the sample (61.2%), had the highest percentage of individuals reporting low distress levels (75.5%), followed by Site 3 (68.4%), Site 1 (63.5%) and Site 4 (61.5%). Site 1 represented 31.9% of the sample. Sites 3 and 4 had smaller sample sizes (4.1% and 2.8%, respectively).

Alcohol use patterns were also significantly associated with psychological distress (*p* < 0.001). Among respondents classified as low risk for alcohol use, the majority (76.5%) reported low distress levels, while 23.5% experienced high psychological distress levels. As alcohol risk increased, the proportion of respondents experiencing high psychological distress levels also increased. For respondents classified as having high risk alcohol use, 46.7% reported high psychological distress levels and for those with likely addiction, 59.1% reported high distress levels.

No statistically significant associations were observed in age, relationship status, work area or work schedule (all *p* > 0.05), suggesting that within this sample, psychological distress is mainly associated with female gender, working at a specific mine site and higher risk alcohol use.

Table 2 indicates the multivariable logistic regression model results, where the outcome variable is psychological distress. Only complete cases of alcohol use patterns, namely 866, were included.

**TABLE 2:** Multivariate logistical regression model (*N* = 866).

Psychological distress	OR	*p*	95% CI		Significant
**Gender**					
Male	1.000	-	-	-	-
Female	1.496	0.027	1.046	2.139	*
**Age (years)**					
< 24	1.000	-	-	-	-
25–34	0.368	0.001	0.206	0.656	*
35–44	0.405	0.004	0.218	0.752	*
45–54	0.356	0.002	0.185	0.686	*
55–65	0.218	0.000	0.095	0.499	*
65 and above	0.381	0.457	0.030	4.830	-
**Relationship status**					
Single	1.000	-	-	-	-
In a relationship (living apart)	1.456	0.170	0.851	2.490	-
In a relationship (Living together)	0.777	0.417	0.423	1.428	-
Separated	1.608	0.403	0.529	4.893	-
Widowed	0.676	0.641	0.131	3.492	-
Married	1.255	0.406	0.734	2.147	-
Divorced	1.946	0.217	0.676	5.599	-
**Work area**					
Office	1.000	-	-	-	-
Plant	0.834	0.407	0.543	1.280	-
Underground	0.855	0.503	0.541	1.353	-
**Work schedule**					
Office hours	1.000	-	-	-	-
Shift hours	0.953	0.804	0.649	1.398	-
**Mine site**					
Site 1	1.000	-	-	-	-
Site 2	0.537	0.000	0.385	0.749	*
Site 3	0.724	0.417	0.333	1.577	-
Site 4	1.017	0.971	0.415	2.489	-
**Alcohol use**					
Low risk	1.000	-	-	-	-
Medium risk	1.763	0.005	1.190	2.611	*
High risk	3.106	0.001	1.631	5.916	*
Addiction likely	5.641	0.000	2.866	11.101	*

Note: 1, Reference variable. *p*-value: Probability > chi^2^.

Significant: *p < 0.05.

OR, odds ratio; Cl, confidence interval.

After controlling for confounding variables, similar variables to the bivariate associations were seen to have a significant association with higher psychological distress, namely female gender, mine site and higher risk alcohol use. Additionally, the association between age and psychological distress was significant.

Gender was a significant factor influencing psychological distress, with females showing statistically higher odds (OR:1.496, *p* = 0.027, 95% CI: 1.046–2.139) than males of higher psychological distress levels.

Age played a significant role, where individuals aged 25–34 years (OR: 0.368, *p* = 0.001, 95% CI: 0.206–0.656), 35–44 years (OR: 0.405, *p* = 0.004, 95% CI: 0.218–0.752) and 45–54 years (OR: 0.356, *p* = 0.002, 95% CI: 0.185–0.686) reported significantly lower psychological distress levels compared to younger age groups (<24 years).

Working at Site 2 was associated with significantly lower odds of distress (OR = 0.537; *p* = 0.00; 95% CI: 0.385–0.749) relative to Site 1, implying a protective effect at Site 2, suggesting that contextual or environmental factors related to the work setting may also contribute to psychological well-being. Sites 3 and 4 did not show significant differences.

Alcohol use also showed significant associations with higher risk categories (Medium risk: OR: 1.763, *p* = 0.005, 95% CI: 1.19–2.611; high risk: OR: 3.106, *p* = 0.001, 95% CI: 1.631–5.916; addiction likely: OR: 5.641, *p* = 0.00, 95% CI: 2.866–11.101) correlating with increased psychological distress levels.

No statistically significant associations existed between relationship statuses, work area, work schedule and psychological distress.

## Discussion

In this study, 29% of participants are likely to have moderate or severe psychological distress. While the K10 is a nonspecific screening tool used widely in epidemiological studies, primary healthcare and other populations of interest,^17^ it screens broadly for symptoms linked to anxiety and depression related to the likely presence of a mental disorder.^18,19^ This finding suggests that 29% of employees could experience moderate to severe anxiety and depressive disorders^17,20^ impacting workplace productivity and general capacity to participate in the workplace.^4,21^

Several studies conducted at mines in Australia and Ghana found lower levels of psychological distress than in the current study, with moderate to severe psychological distress ranging between 13% and 15% in these studies.^5,7,22,23^ Considine et al.^24^ and Bowers et al.^25^ showed similar findings to the current study, with 29% of the participants in mining and construction in Australia having moderate to severe psychological distress. A South African study conducted in the primary healthcare environment had similar findings, with 31% of respondents having moderate to severe psychological distress.^26^

The findings from a review by the Centre for Transformative Work Design,^27^ considering studies conducted between 1998 and 2022, conclude that mental health and well-being in mining are generally poorer than in other industries. However, there are some mixed findings, with other studies showing similar or better mental health.^28^ This report further indicates that levels of distress have increased in the past 6 years.^27^

Similar to this study’s findings, James et al.^7^ found that an interplay of personal, workplace and social factors was associated with psychological distress in the mining environment. A multitude of societal, organisational and individual factors affect the mental health of employees.^29^

The results of this study, suggesting that women are more likely to experience psychological distress than men, are similar to several other population-based and mining-specific studies.^30,31,32,33^ Despite interventions, protocols and legislation developed to provide equal opportunities for women in mining, they still experience social, physiological, structural and employment barriers that may affect their mental health. These barriers include challenges with career advancement, gender stereotypes, exposure to organisational and interpersonal sexism, isolation and difficulty in balancing work and household responsibilities.^33,34,35,36,37,38,39^ The model of continuous production in mining leads to prolonged and intense working hours, that makes it difficult for women to combine their work and family caretaker roles.^37,39^ Considering these barriers, management of mines should create interventions, policies and procedures that increase physically and psychologically safe environments for women.^36^

The finding about age suggests that early adulthood may be a vulnerable period for psychological distress. Similar to the current study, other studies found that the rates of high psychological distress decreased with age.^5,7,25^ Younger employees experience higher psychological distress, possibly due to their relative inexperience in coping with work requirements and demands and the stressors associated with having young families with childcare responsibilities.^40,41^ Coping styles mediate the effect of psychological distress with healthier coping styles linked to lower levels of psychological distress.^42,43^

Mine Site 2 was associated with lower psychological distress than Site 1. Sites 1 and 2 are similar in size and the type of operations involved in underground mining. Environmental and workplace differences between the two sites likely contribute to the differences. Site 1 is in an urban area, in a province with a decrease in mining employment statistics, and Site 2 is in a semi-rural to rural environment in a province showing an increase in mining employment statistics in the third quarter of 2022 and 2023. A decrease in employment statistics suggests there is potential job insecurity for miners in those provinces.^44^ Job security is linked with psychological distress, with more psychological distress experienced where the likelihood of job loss is greater.^7^ Social and managerial support may differ at the two sites, with higher social and managerial support associated with lower levels of psychological distress.^5,7,23,24^ Based on the evidence that life stressors and life events impact on community mental health, it is possible to assume that community factors in these two environments further impact on workers’ mental health.^7^

There is a strong link between alcohol use and mental health. Co-occurring alcohol use disorders and mental disorders are highly prevalent and are linked to heightened risk to self and others, greater psychological and physical impairment and poorer prognosis.^45^ Similar to the findings of this study, there is a well-recognised positive relationship between hazardous alcohol use and psychological distress in general population and mining specific studies.^5,7,23,46,47,48^ The relationship between alcohol use and mental health is, however, complex and likely connected through multiple pathways. These pathways are direct (alcohol use affects mental health), bidirectional (mental health affects alcohol use and alcohol use affects mental health), or have factors affecting both, for example, stress affecting alcohol use and psychological distress.^49,50^

### Study limitations

Self-report bias, such as desirability bias and recall errors could have affected the findings. Data about the missing cases are not included in the current analyses. Despite a larger than required sample size (also for each of the four subsites) and response rate of 84%, the missing data introduce bias into the findings as persons with higher psychological distress, or those with higher alcohol use patterns may have been more likely to submit incomplete responses. Due to the nonprobability sampling method used and the over-representation of participants from Site 2, generalisability is limited. Distribution of complete cases mirrored the actual sample confirming data loss was relatively even across sites.

## Conclusion

This study provides evidence that personal, workplace and lifestyle factors influence psychological distress at the mining company. Twenty-nine per cent of the respondents reported high levels of psychological distress, suggesting a third of the workforce is at potential risk for adverse mental health outcomes affecting their ability to contribute to the work environment optimally. The combined analyses from the bivariate and multivariable models suggest that psychological distress among the study population is significantly associated with gender, age, mine site and alcohol use.

The findings of the current study are consistent with the previous studies that have documented higher psychological distress among females, younger employees and those engaging in risky alcohol use. Identifying mine site-specific differences adds a new dimension to the current understanding, suggesting that environmental factors and additional work-related factors may be associated with psychological distress. This insight encourages a broader view of workplace mental health, integrating environmental context into existing frameworks that require further investigation.

Based on these findings, we recommend that targeted mental health interventions be prioritised for high-risk groups such as females, young adults and individuals with elevated alcohol consumption. Integrated interventions addressing personal, work, lifestyle and environmental factors will likely benefit individuals and the workplace. Enhancing workplace conditions through site-specific strategies could further contribute to mitigating psychological distress. The findings highlight the importance of monitoring alcohol use as a factor in mental health.

Future research should explain these associations’ causal mechanisms through longitudinal and experimental studies and explore the lived experiences of women and younger employees to provide context-relevant interventions. Intervention studies assessing the impact of targeted mental health strategies in the mining company could further their efficacy and inform evidence-based policy-making and resource allocation.
